# Emerging Roles of Immune Cells in Postoperative Cognitive Dysfunction

**DOI:** 10.1155/2018/6215350

**Published:** 2018-02-18

**Authors:** Yue Liu, Yiqing Yin

**Affiliations:** Department of Anesthesiology, China-Japan Friendship Hospital, Beijing 100029, China

## Abstract

Postoperative cognitive dysfunction (POCD), a long-lasting cognitive decline after surgery, is currently a major clinical problem with no clear pathophysiological mechanism or effective therapy. Accumulating evidence suggests that neuroinflammation plays a critical role in POCD. After surgery, alarmins are leaked from the injury sites and proinflammatory cytokines are increased in the peripheral circulation. Neurons in the hippocampus, which is responsible for learning and memory, can be damaged by cytokines transmitted to the brain parenchyma. Microglia, bone marrow-derived macrophages, mast cells, and T cells in the central nervous system (CNS) can be activated to secrete more cytokines, further aggravating neuroinflammation after surgery. Conversely, blocking the inflammation network between these immune cells and related cytokines alleviates POCD in experimental animals. Thus, a deeper understanding of the roles of immune cells and the crosstalk between them in POCD may uncover promising therapeutic targets for POCD treatment and prevention. Here, we reviewed several major immune cells and discussed their functional roles in POCD.

## 1. Introduction

Postoperative cognitive dysfunction (POCD) refers to a long-lasting cognitive decline after surgery, characterized by impaired concentration, memory, and learning, which can be detected by a battery of neuropsychological tests [1]. The incidence of POCD is 7 to 26% after major noncardiac surgery and even higher in patients older than 60 years [1, 2]. POCD not only diminishes the patient's quality of life and imposes a serious burden on healthcare costs but also increases mortality [3]. Although several risk factors for POCD have been identified, the pathophysiological mechanism underlying POCD remains unclear and no effective therapies have been developed to date.

A large number of studies conducted in patients have revealed that POCD is associated with elevated levels of plasma inflammatory cytokines, including tumor necrosis factor- (TNF-) *α* and interleukin- (IL-) 6 [4–7]. IL-1*β* and IL-6 levels in the cerebrospinal fluid (CSF) of patients with POCD are higher than those of patients with normal cognitive function after surgery [8, 9]. The learning and memory function was also impaired by surgery and anesthesia in experimental animals, accompanied by the upregulation of proinflammatory cytokine levels in both the blood and the brain [10–13]. Neuroinflammation, particularly in the hippocampus, has been proved to be one of the main causes of POCD [14–17]. The activation of microglia and other blood-derived immune cells orchestrates neuroinflammation and subsequent neuronal damage [14–17]. In this review, we discuss the main types of immune cells involved in POCD and their possible roles. We describe their functions in neuroinflammation, put forth a possible mechanism of their involvement in POCD, and point out the fields that need further exploration.

## 2. Immune Cells in POCD

### 2.1. Microglia

Microglia are highly specialized tissue-resident macrophages in the central nervous system (CNS) and the major resident immune cells of the brain [18]. Microglia are the only CNS cells originating from hematopoiesis. Primitive macrophage progenitors in the yolk sac colonize the CNS and differentiate into mature microglia, confined behind the blood-brain barrier (BBB) [19]. Unlike other tissue macrophages, such as Kupffer cells in the hepatic sinusoids, which need to be renewed from bone marrow progenitors, microglia are capable of local expansion and maintenance throughout life without reconstitution from the bone marrow [20].

In healthy brains, microglia are ramified and in a resting state, monitoring the local microenvironment and detecting CNS damage [21]. Danger signals, including pathogen invasion, injury, and abnormal protein accumulation, can trigger microglia transformation into an activated amoeboid shape. Activated microglia are both neuroprotective and neurotoxic. Studies in adult and neonatal hypoxic-ischemic injury models have shown that a complete blockade of microglial activity exacerbates brain damage [22, 23]. However, activated microglia can also produce excessive proinflammatory cytokines, leading to neuronal dysfunction and death. Several neurodegenerative diseases, including Alzheimer's, Huntington's, and Parkinson's diseases, have been proved to be associated with the hyperactivation of microglia [24–26]. As a specific type of macrophages, activated microglia can have one of the two different phenotypes: classically activated M1 and alternatively activated M2 microglia. M1 microglia promote inflammation by secreting proinflammatory cytokines such as IL-1*α*, IL-1*β*, and TNF. M2 microglia elicit neuroprotective effects through the release of vascular endothelial growth factor and extracellular matrix proteins [27]. In Alzheimer's disease (AD), amyloid *β* (A*β*) sensitizes microglia to subsequent cytokine stimulation and M1 activation [28], whereas the induction of the M2 polarization of microglia by drugs or adeno-associated viral vectors can reduce A*β* deposition and relieve AD symptoms [29, 30]. In other neurological diseases, such as Parkinson's disease (PD), chronic cerebral hypoperfusion, traumatic brain injury, and hepatic encephalopathy, the priming of microglial polarization towards the M1 phenotype plays a pivotal role in neuroinflammation [31–34].

After peripheral surgery, an immune challenge is transmitted to the brain via multiple humoral and neural routes. The integrity of the BBB can be disrupted by a systemic inflammatory response or anesthesia during and after surgery [11, 35, 36]. Adenosine triphosphate (ATP), alarmins, and cytokines, which are leaked from an injury site or increase in response to systemic inflammation, enter the brain and activate microglia [11, 36–38]. Activated microglia may impair learning and memory via the release of proinflammatory cytokines, among which IL-1*β* and TNF-*α* are particularly important [38, 39]. Mild repeated stress or systemic endotoxin challenge can trigger microglia to secrete IL-1*β* and TNF-*α* [38, 40–42]. After surgery, aged rats and mice demonstrated significant deficits in memory and learning, concurrent with the activation of microglia and increased expression of TNF-*α* and IL-1*β* in the hippocampus [43, 44]. Preemptively depleting microglia reduced surgery-induced hippocampal inflammatory cytokine secretion and attenuated the cognitive decline in mice [14]. IL-1*β* and TNF-*α* can cause neuronal cell death, reduction of acetylcholine release, and attenuation of glutamatergic transmission, resulting in learning and memory deficits [38, 40–42]. Neuroinflammation and POCD were mitigated in IL-1R knockout mice or mice pretreated with an IL-1 receptor (IL-1R) antagonist compared with control mice [10]. Furthermore, microglia can be activated by peripheral TNF-*α* [38]. Preemptive treatment of anti-TNF antibody is able to limit the release of IL-1 in the hippocampus and prevent cognitive decline in a mouse model of POCD [11]. Therefore, microglia may respond to peripheral TNF-*α*, secrete more TNF-*α* and IL-1*β* in the hippocampus, and amplify neuroinflammation in POCD. Additionally, a study also reported reduced infiltration of bone marrow-derived monocytes into the hippocampus after microglial depletion, suggesting crosstalk between microglia and bone marrow-derived macrophages (BMDMs) in POCD [14].

No studies to date have reported the polarization of microglia in POCD. However, the main cytokines secreted by activated microglia in POCD are IL-1 and TNF-*α* [14, 43, 44], suggesting the predominance of the M1 state of microglia in POCD. Furthermore, the M2 response of microglia was impaired after brain ischemia in aged mice [45]. Because older patients are particularly susceptible to POCD, we speculate that the M1 phenotype of microglia plays a central role in neuroinflammation in POCD. Pharmacological approaches that have been successfully used to modulate microglia polarization in other neurological diseases may hold promise for developing POCD treatments [32, 34].

In a synthesis of the existing microglia and POCD research, we can draw a picture of how microglia may orchestrate postoperative neuroinflammation in POCD. As the resident immune cells of the brain parenchyma, microglia are activated by proteins and other signals leaked from the injury sites. The cytokines secreted from the microglia can directly damage neurons and also recruit more immune cells from the blood penetrating into the brain parenchyma, further accelerating neuronal injury.

### 2.2. Bone Marrow-Derived Macrophages

Macrophages are present in virtually all tissues. They differentiate from circulating peripheral-blood mononuclear cells, which migrate into tissues constitutively or in response to inflammation [46]. In a healthy CNS, BMDMs are divided into three classes according to their location: choroid plexus, meningeal, and perivascular macrophages [20]. These macrophages are exterior to the brain parenchyma, and their population homeostasis is achieved by replacement from blood-born monocytes. In disease states, BMDMs respond to inflammation and migrate into the brain parenchyma from the circulation.

BMDMs are a major component of the inflammatory immune response to CNS diseases. Similar to microglia, BMDMs have a proinflammatory M1 phenotype and an anti-inflammatory M2 phenotype. M2 macrophages can be beneficial for the healing of sterilized wounds, clearing necrotic debris or abnormal proteins. In a spinal cord injury model, macrophages played an anti-inflammatory role during recovery [47]. Furthermore, numerous studies have suggested that BMDMs can infiltrate the brain, reduce the A*β* plaque burden, and alleviate the cognitive decline in AD [48, 49]. In a clinical study, transplantation of autologous M2 macrophages significantly improved motor and cognitive activities in patients with severe cerebral palsy [50]. Other reports, however, have indicated that macrophages mainly play a detrimental role in CNS pathology. Penetration of macrophages into the brain impaired spatial learning and memory after traumatic brain injury in mice [51, 52]. In a model of intracerebral hemorrhage, mice exhibited improved motor function after the depletion of inflammatory monocytes [53]. In addition, circulating monocytes or macrophages have been implicated in the exacerbation and relapses of experimental autoimmune encephalitis (EAE) in mice [54, 55].

BMDMs were found in the hippocampi of mice with POCD [56]. Depletion of BMDMs attenuated surgery-induced increases of the IL-6 levels in serum and the hippocampus, reduced hippocampal macrophage infiltration, and prevented surgery-induced memory dysfunction [15]. Inhibiting the proinflammatory signaling pathway in BMDMs or preserving the integrity of the BBB can also reduce the infiltration of BMDMs in the hippocampus and prevent POCD [56]. Furthermore, mice deficient in IL-6 exhibited less IL-1*β* and TNF-*α* expression in the hippocampus and better working memory [57]. These findings indicate that, with the BBB integrity disrupted, BMDMs infiltrate into the hippocampus and secrete proinflammatory cytokines, exacerbating neuroinflammation in POCD.

The depletion of microglia has also been shown to prevent BMDMs infiltrating the hippocampus without impairing the capacity of monocytes to penetrate into the brain [14]. Monocyte chemotactic protein-1 (MCP-1), also known as CCL2, is a major chemoattractant to recruit BMDMs [58]. Postoperative hippocampal MCP-1 levels were reduced by the depletion of microglia [14] but not BMDMs [15], indicating that microglia are the major source of secreted MCP-1. Taken together, these studies show that microglia attract BMDMs into the brain via MCP-1 secretion after injury.

High-mobility group box 1 protein (HMGB1), a ubiquitous nucleosomal protein, is passively released into the circulation from damaged necrotic cells, and circulating HMGB1 levels increase after surgery [36, 59]. Blocking the HMGB1 function with a monoclonal antibody reduced the hippocampal expression of MCP-1 and postoperative memory decline in mice [60]. Furthermore, the depletion of BMDMs prevented an HMGB1-mediated memory decline after surgery [60]. Together with the previous studies, these results indicate that HMGB1 may stimulate hippocampal microglia to secrete MCP-1, enabling monocyte recruitment. Similar to HMGB1, many cytokines can simulate microglia. In a model of peripheral organ inflammation, microglia were stimulated by peripheral TNF-*α* and attracted circulating monocytes into the brain [61]. Moreover, plasma TNF-*α* levels were upregulated early after aseptic surgery, and a blockade of TNF-*α* prevented POCD in mice [11]. However, whether the TNF-*α*/microglia/BMDM pathway is essential in the pathogenesis of POCD is still unknown.

In summary, the activation of microglia and BMDM recruitment play important roles in POCD. However, the relationship between microglia and BMDMs in POCD needs further investigation. The possibility of BMDM infiltration into the CNS after surgery through other microglia-independent pathways also needs exploration.

### 2.3. Mast Cells

Mast cells (MCs) are myeloid cells originating from CD34^+^/CD117^+^ pluripotent progenitor cells [62]. MCs contain many cytoplasmic granules, which store a number of preformed mediators, including histamine, heparin, serotonin, chymase, tryptase, prostaglandins, and leukotrienes. MCs are best known for their roles in allergic disease and host defense. Crosslinking immunoglobulin E (IgE) receptors of MCs triggers the release of many allergic and inflammatory mediators [63]. MCs are abundant within tissues exposed to the external environment, such as the skin, gut, and the respiratory tract. MCs are also present in the CNS, mainly located in the perivascular spaces and along the leptomeninges [64, 65]. Upon activation, MCs can release the mediators and infiltrate into the brain parenchyma, participating in the pathophysiological processes of various neurological diseases.

It is well established that MCs contribute to general vascular permeability through the production of vasodilators, such as histamine and serotonin. Ample evidence also exists that the vasodilatory and proinflammatory mediators released by MCs contribute to the impairment of the BBB integrity (reviewed in [66]). For instance, histamine can open the tight junctions between the endothelia in the BBB [67]. Proteinases secreted by MCs, including tryptase and gelatinase, can degrade protein constituents of the neurovascular matrix, thus damaging the BBB [67]. In recent decades, studies have demonstrated that MCs play critical roles in the disruption of the BBB and associated neurological diseases. Acute stress increased the permeability of BBB through the activation of MCs [68]. Furthermore, compared with wild-type mice, MC-deficient mice showed decreased BBB permeability, reduced T cell infiltration, and, consequently, less severe EAE [69]. In addition, in a mouse model of brain ischemia, animals that were deficient in MCs or treated with the MC stabilizer Cromolyn exhibited improved BBB integrity and reduced brain edema [70].

Studies have suggested that MCs are the predominant cells that initiate glial activation. In a model of perinatal hypoxia-ischemia, MCs were found to be the “first responders,” with their activation preceding that of microglia [71]. In addition, the clinical conditions of depression and mild neurocognitive disorders are closely related to the malfunction of the MC-glia crosstalk [72]. Microglia express a large variety of proteins/receptors that can be activated by MC-secreted mediators. For instance, tryptase can trigger microglia activation through the proteinase-activated receptor 2 (PAR2) [73]. Furthermore, microglia express all four histamine receptors (HRs) and can be activated by MCs via HRs [74, 75]. Astrocytes also express PAR2 and HRs and can be activated by MCs [76, 77]. The interactions between MCs and glial cells are not restricted to the receptors mentioned above (reviewed in [78]), and accumulating evidence indicates that MCs and glial cells work in concert to promote neuroinflammation [78].

While numerous studies in rodents have explored the role of MCs in neurological diseases, relatively few have focused on MC function in POCD. Surgery was found to induce MC degranulation in mice [79]. Rats treated with the MC stabilizer Cromolyn showed less severe cognitive deficits after surgery, accompanied by increased BBB stability [16] and reduced microglia and astrocyte activation [79, 80]. Therefore, via disrupting BBB and activating microglia, MCs promote neuroinflammation in POCD. In the studies of MCs in POCD, Cromolyn was administered intracerebroventricularly [16, 79, 80]; the therapeutic efficacy of Cromolyn administered via other routes remains to be established. Masitinib, an oral selective tyrosine-kinase inhibitor, can effectively inhibit the survival, migration, and activity of MCs. In a clinical trial, masitinib slowed the cognitive decline in patients with AD [81]. The effectiveness of masitinib in the treatment and prevention of POCD also needs further investigation.

### 2.4. T Cells

The thymus-derived T cells constitute key players in antigen-specific immune responses. T cells are divided into three main functional subsets: CD8 cells, also known as cytotoxic T cells; helper CD4 cells (Th cells); and regulatory CD4 cells (Treg cells). In healthy noninflamed CSF, 90% of the total cells are T cells, predominantly CD4 cells [82]. In a pathological state, T cells can penetrate into the brain parenchyma. Multiple studies have shown the importance of T cells in autoimmune and virus infectious neurological diseases, such as multiple sclerosis and herpes simplex virus encephalitis [83]. Recently, the roles of T cells in neurodegenerative diseases have also received much attention. The activation of Th cells enhances the loss of dopaminergic neurons in a mouse model of PD [84], while Treg cells provide neuroprotection through the attenuation of microglial activation in this disorder [85].

There is no direct evidence of T cells participating in the pathological process of POCD. One study demonstrated that surgery-induced cognitive impairment in mice was accompanied by upregulation of IL-17 and downregulation of IL-10 expression, mainly in Th17 (a subset of Th cells) and Treg cells, respectively [17]. This study proposed the possibility that a T cell-subtype imbalance may contribute to POCD. More evidence is needed to uncover the role of T cells in POCD.

## 3. Conclusion

**Figure 1:**
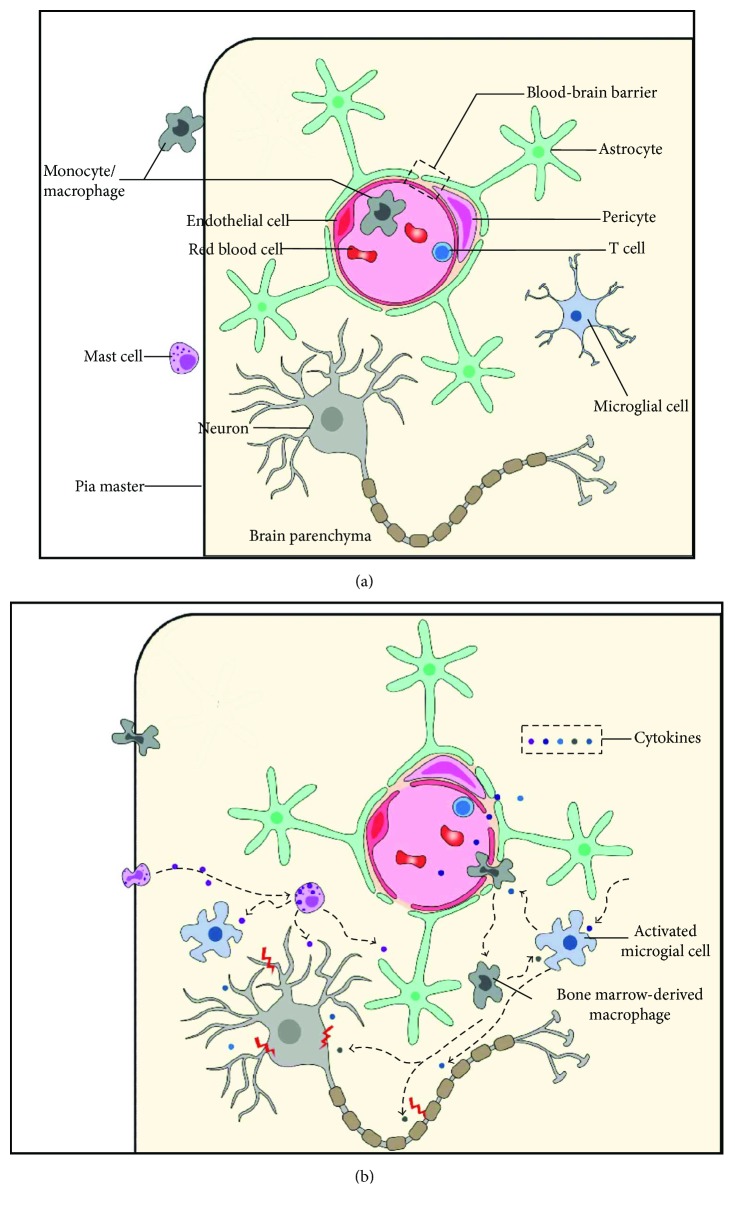
Schematic diagram of immune cells in POCD. (a) Under a normal condition, neurons are normally functioning. Microglia are ramified and in a resting state. The BBB is intact. Monocytes, mast cells, and T cells are restricted outside the brain parenchyma. (b) After surgery, many cytokines are released from the injured sites and damage the BBB. Microglia are triggered by these cytokines and turned into an activated, amoeboid shape. Microglia-secreted cytokines can damage neurons and also recruit BMDMs and other inflammatory cells from the blood. BMDMs and MCs infiltrate into the brain parenchyma and release more cytokines, which can directly damage neurons and also activate microglia. Cytokines secreted by T cells also participate in neuroinflammation in POCD. The immune cells and cytokines compose an inflammation network that aggravates neural damage, leading to POCD. POCD: postoperative cognitive dysfunction; BBB: blood-brain barrier; BMDM: bone marrow-derived macrophage; MC: mast cell.

While a plethora of studies have suggested that immune cells trigger neuroinflammation in response to surgery leading to POCD, the neurobiological basis of POCD remains unknown (Figure 1). As the major resident immune cells in the CNS, microglia are activated by proteins released from the injury sites and circulating cytokines upregulated by surgery. The activation of microglia results in neuronal damage via the release of proinflammatory cytokines. Circulating BMDMs are recruited into the brain in response to surgery, a process that may be initiated by microglia-secreted MCP-1. The degranulation of MCs contributes to BBB disruption and the activation of microglia, further aggravating POCD. T cells may also be involved in POCD.

These immune cells interact with one another in the pathogenesis of POCD. Different elements of the resulting network of neuroinflammation may serve as targets in the prevention and treatment of POCD. First, cytokines leaking from the injury site are the primary trigger of the immune response in the CNS. Thus, approaches that inhibit cytokine release may prevent POCD. Second, microglia occupy the central position of the inflammatory network; hence, drugs that stabilize microglia or promote their transition to the M2 state may have beneficial effects. Third, other circulating immune cells penetrating into the brain parenchyma and secreting inflammatory cytokines exacerbate neuroinflammation. Therefore, therapies that reduce cytokine secretion by these immune cells may also be effective for treating POCD. Studies in rodents using blocking antibodies and other agents interfering with the neuroinflammation network have provided proof of concept for these strategies as POCD treatments [10, 11, 15, 17, 56, 60, 79, 80]. However, their feasibility in humans still needs to be validated. Further research on the mechanisms of immune cell involvement in POCD is urgently required to identify other potential targets for POCD treatment and prophylaxis.
